# Relationship of pre-diagnosis pharmacist continuity with patient-reported satisfaction at the first post-diagnosis community pharmacy visit among adolescents and young adults with cancer: a nationwide cross-sectional online survey

**DOI:** 10.1186/s40780-026-00589-9

**Published:** 2026-06-09

**Authors:** Atsunobu Sagara, Tomofumi Watanabe, Tomoya Abe, Hiroyuki Terakado

**Affiliations:** 1 Department of Clinical Epidemiology, School of Pharmacy and Pharmaceutical Sciences, Hoshi University, 2-4-41 Ebara, Shinagawa-ku, Tokyo, Japan; 2 Department of Pharmacy, Japanese Red Cross Medical Center, 4-1-22 Hiroo, Shibuya-ku, Tokyo, Japan; 3 Department of Pharmacy, Saitama Cancer Center, 780 Komuro, Ina- machi, Kitaadachi-gun, Saitama, Japan

**Keywords:** Adolescents and young adults, Pharmacist continuity, Patient-reported satisfaction, Community pharmacy, MISS-21J

## Abstract

**Background:**

Adolescents and young adults (AYAs) with cancer often face developmental stage–specific challenges related to education and employment, interpersonal relationships, fertility, and family formation. Psychosocial support is important in cancer care, especially since psychological distress and anxiety tend to increase immediately after diagnosis. Some patients have an established relationship with a community pharmacist before diagnosis, but it remains unclear whether such continuity is associated with patient-reported satisfaction with pharmacist consultations during the early post-diagnosis period. Using nationwide survey data, this study investigated the association between patient-reported satisfaction at the first community pharmacy visit after an initial cancer diagnosis, assessed using the Medical Interview Satisfaction Scale Japanese version (MISS-21J), and pre-diagnosis pharmacist continuity.

**Methods:**

A nationwide cross-sectional online survey was conducted from March 25 to 27, 2025. Data were analyzed for 407 patients with an initial cancer diagnosis who were diagnosed at age 15–39 years and visited a community pharmacy after diagnosis. Participants were grouped according to the presence or absence of pre-diagnosis pharmacist continuity. The prespecified primary outcomes were the MISS-21J distress relief and rapport subscale scores, which reflect domains of patient-reported satisfaction with pharmacist consultations. Multivariable linear regression models (ordinary least squares) were fitted, adjusting for age at diagnosis, sex, cancer type, residential area, parents available, partner status, and minor children. Adjusted means (least-squares means; LSMeans) and adjusted mean differences with 95% confidence intervals (CIs) were reported.

**Results:**

For distress relief, the adjusted mean was 5.33 in patients with pre-diagnosis pharmacist continuity and 4.72 in those without such continuity (adjusted mean difference: 0.61; 95% CI, 0.42–0.81; *p* < 0.001). For rapport, the adjusted mean was 5.20 in patients with pre-diagnosis pharmacist continuity and 4.60 in those without such continuity (adjusted mean difference: 0.60; 95% CI, 0.41–0.80; *p* < 0.001).

**Conclusions:**

Among AYAs newly diagnosed with cancer, pre-diagnosis pharmacist continuity was associated with higher patient-reported satisfaction with pharmacist consultations, particularly in the MISS-21J domains of distress relief and rapport. These findings highlight the potential value of accessible, continuous relationships with pharmacists in improving patients’ perceived pharmaceutical care experience during the early post-diagnosis period.

**Supplementary Information:**

The online version contains supplementary material available at 10.1186/s40780-026-00589-9.

## Background

Adolescents and young adults (AYAs) with cancer represent a distinct population with age-specific psychosocial challenges [1–3]. These patients typically experience cancer during critical life transitions—such as education and employment, interpersonal relationships, fertility, marriage and family formation, and parenting—and may therefore face unique burdens beyond disease management [4–6]. Accordingly, international and domestic frameworks have emphasized the importance of incorporating developmentally appropriate, multidisciplinary psychosocial support into AYA cancer care [7–9]. However, because AYAs are often healthy prior to diagnosis, routine contact with healthcare resources, including community pharmacies, may be limited.

Being diagnosed with cancer is a major life event that can rapidly cause psychological distress, anxiety, and uncertainty regarding treatment and daily life, especially during the period immediately after diagnosis [10, 11]. However, it is not always easy to provide timely, patient-centered support that adequately addresses these early needs [12, 13]. As a result, even if problems emerge soon after diagnosis, patients may have limited access to readily available sources of consultation. Community pharmacies and pharmacists are readily accessible healthcare resources who can provide medication counseling, management of adverse effects, symptom management, responses to treatment-related concerns, and coordination with other healthcare professionals during the early post-diagnosis period [14].

In Japan, the kakaritsuke-yakuzaishi (family pharmacist) system was introduced in 2016, enabling patients to designate a specific pharmacist and receive ongoing consultation and support [15–17]. An established relationship with a pharmacist prior to diagnosis may facilitate help-seeking and contribute to more favorable patient perceptions of pharmacist consultations when treatment-related concerns arise after diagnosis. However, further examination is needed to clarify whether continuity with a pharmacist before the initial cancer diagnosis is associated with patient-reported satisfaction with pharmacist consultations during the early post-diagnosis period among AYAs.

The Medical Interview Satisfaction Scale (MISS) is a quantitative measure of patient satisfaction with medical interviews [18]. The Japanese version, MISS-21J, has demonstrated reliability and validity for evaluating pharmacist-provided patient support and assesses multiple dimensions of satisfaction [19], including distress relief, communication comfort, rapport, and compliance intent. In the original validation study, the MISS-21J showed acceptable-to-high internal consistency for its four subscales, with Cronbach’s alpha values of 0.906 for distress relief, 0.759 for communication comfort, 0.915 for rapport, and 0.800 for compliance intent [19]. Although the MISS-21J was originally validated in the context of pharmacist counseling for over-the-counter medication, its subscales assess general domains of pharmacist consultation experience, such as perceived relief, communication comfort, interpersonal rapport, and intention to follow medication-related advice. Therefore, we considered it a reasonable available instrument for assessing patient-reported satisfaction with pharmacist consultations in the present study, while recognizing that the prescription medication and cancer care context differs from the original validation setting and that further validation in AYA oncology settings is warranted.

Using nationwide survey data, this study examined the association between pre-diagnosis pharmacist continuity and patient-reported satisfaction with pharmacist consultations, assessed using the MISS-21J, at the first community pharmacy visit following the initial cancer diagnosis among AYAs. Because the MISS-21J distress relief and rapport domains reflect patients’ perceived relief and trust within the consultation experience, which were considered particularly relevant to early post-diagnosis consultations, these subscales were prespecified as the primary outcomes. The remaining subscales were evaluated secondarily in an exploratory manner.

## Methods

### Survey period and participants

A nationwide cross-sectional online survey was conducted from March 25 to 27, 2025 using a large web-based research panel maintained by Macromill, Inc. The survey was conducted as a closed online survey administered to registered Macromill panel members through the vendor’s standard survey distribution system. The survey was not publicly accessible through an open URL and could be answered only by registered panel members who were invited by the vendor. At the time of the survey, the relevant Macromill panels included 1,410,501 registered members in total, comprising 5,959 members registered as cancer patients based on self-reported information and 1,404,542 members in a general panel without registered information on past medical history. The preliminary screening survey was distributed to 334,211 panel members, including 5,959 members from the cancer patient panel and 328,252 members from the general panel. A total of 22,850 respondents answered the preliminary screening survey, including 1,066 respondents from the cancer patient panel and 21,784 respondents from the general panel. The preliminary screening survey was used to identify respondents who were potentially eligible by assessing items including self-reported history of cancer diagnosis and age at initial cancer diagnosis. After the preliminary screening process, 2,165 respondents were invited to the main survey, and 1,663 respondents completed the main survey before vendor data-quality screening. After excluding 230 responses judged by the vendor to be inappropriate based on data-quality screening, including extremely short response times and inconsistent responses, 1,433 completed responses were included in the delivered dataset.

The survey included participants who (1) were diagnosed with cancer for the first time between age 15–39 years, (2) had undergone cancer treatment, and (3) were aged 18–49 years at the time of the survey. AYAs were defined as patients who were aged 15–39 years old at the initial cancer diagnosis, as per the commonly used definition [9]. In this manuscript, “initial cancer diagnosis” refers to the first diagnosis of cancer reported by the respondent. The term “primary site” refers to the cancer site at the initial cancer diagnosis.

**Fig. 1:**
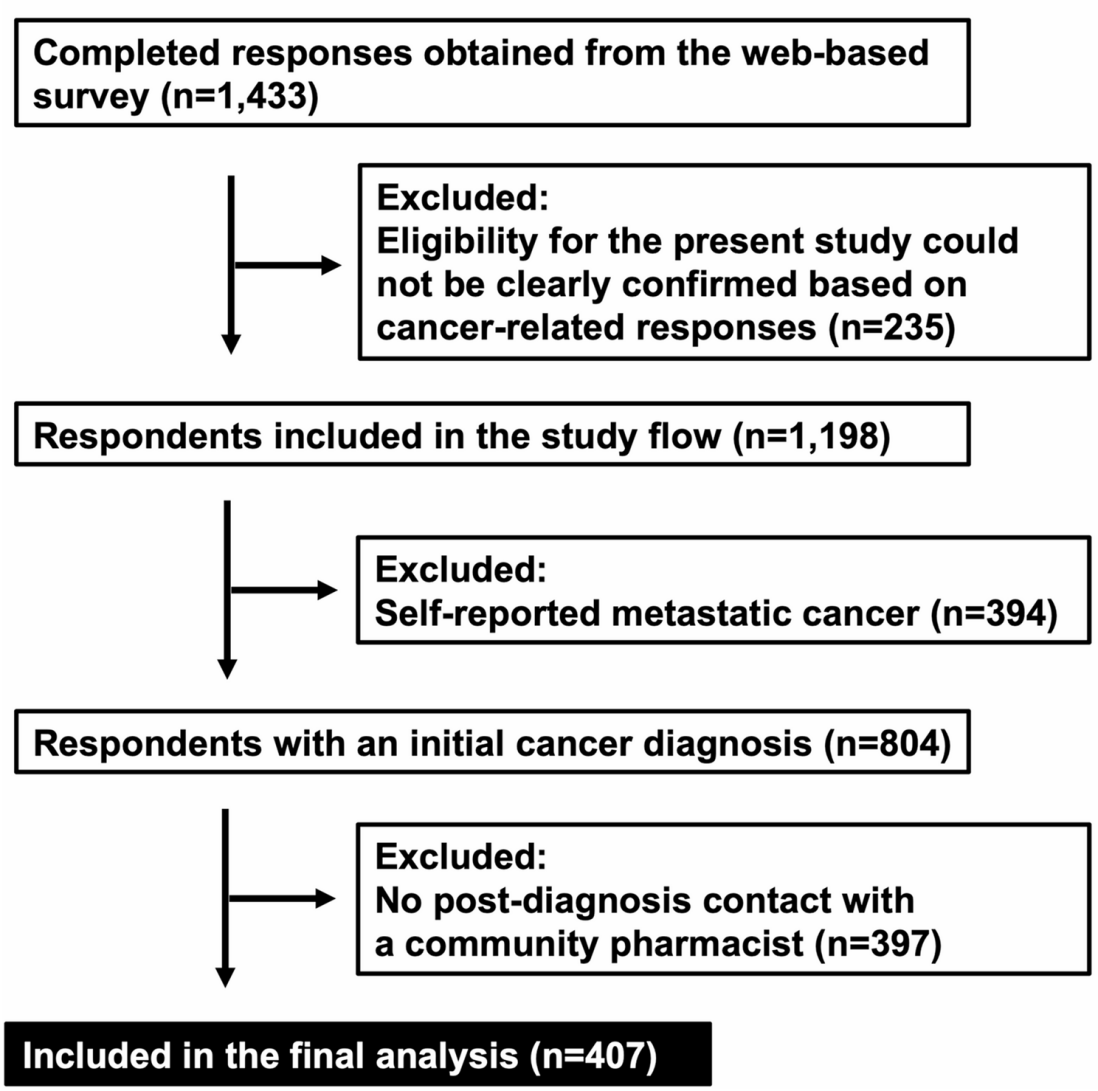
Participant flow. A total of 1,433 completed responses were obtained from the web-based survey. After excluding 235 respondents whose eligibility for the present study could not be clearly confirmed based on cancer-related responses, 1,198 respondents were included in the study flow. Among these respondents, 394 self-reported metastatic cancer and were excluded. Among the remaining 804 respondents with an initial cancer diagnosis, 397 had no post-diagnosis contact with a community pharmacist and were excluded. The final analysis included 407 respondents

The present analysis focused on the first community pharmacy visit following the initial cancer diagnosis. The respondents were those who (i) visited a community pharmacy and had contact with a pharmacist, as assessed by the questionnaire item on routine dispensing and counseling, and (ii) self-reported an initial cancer diagnosis. Respondents who self-reported having metastatic cancer were excluded. This restriction was applied to focus on the post-diagnosis pharmacy visit following the initial diagnosis and to minimize potential recall contamination by later experiences. After reviewing cancer-related responses and excluding 235 respondents whose eligibility for the present study could not be clearly confirmed based on cancer-related responses, 1,198 respondents were included in the study flow. Among these respondents, 394 self-reported metastatic cancer and were excluded, leaving 804 respondents with an initial cancer diagnosis. Among these 804 respondents, 407 had a post-diagnosis community pharmacy visit with pharmacist contact, whereas 397 had no contact with a community pharmacist. The final analysis included 407 respondents (Fig. 1).

The survey system allowed only one response per registered monitor ID, thereby preventing duplicate responses from the same panel member. After data receipt, the investigators checked the anonymized monitor IDs, and no duplicate IDs were identified. All questionnaire items used in the present analysis were mandatory; therefore, no item-level missing data were present in the analytic dataset, and no imputation was performed. Information on mean or median response time was not available to the investigators. As a prerequisite for data cleaning, the survey used panels that had undergone the vendor’s routine panel-quality management procedures, including monthly inactive-member checks, semiannual trap surveys to identify inappropriate respondents, and mandatory annual updates of registered monitor information [20, 21]. Participants received proprietary reward points issued by the survey vendor as compensation for survey completion. Reporting of this web-based cross-sectional study was guided by the STROBE statement for cross-sectional studies and, where applicable, the CHERRIES checklist for web-based surveys.

### Survey items

All survey items and response options are shown in Supplementary Table S1. Sex and residential area were obtained from the basic registration information recorded by Macromill, Inc. Age at diagnosis was self-reported and collected using single-year response options (15 to 39 years). Cancer type (primary site) was self-reported using a predefined list (single response). For analysis and presentation, cancer types with fewer than 15 participants in the total sample were combined under the “Other” category to avoid sparse categories in descriptive and regression analyses. Malignant lymphoma and leukemia were combined under “hematologic malignancies.” Social background variables were assessed as binary items (yes/no), including availability of parents when needed, partner status, and presence of minor children.

To reflect the terminology used in Japan’s healthcare system, the questionnaire used the term “kakaritsuke-yakuzaishi” (family pharmacist). Participants were asked whether they had a family pharmacist before their initial cancer diagnosis and whether they were supported by the same pharmacist at their first community pharmacy visit shortly after diagnosis. For the purposes of analysis, however, the main exposure was operationally defined as pre-diagnosis pharmacist continuity, indicating continuity with the same community pharmacist from before the initial cancer diagnosis to the first post-diagnosis pharmacy visit. Participants were classified into two groups: (i) with pre-diagnosis pharmacist continuity, defined as having a family pharmacist before diagnosis and being served by the same pharmacist at the first post-diagnosis pharmacy visit; and (ii) without pre-diagnosis pharmacist continuity, defined as not meeting this criterion.

Patient-reported satisfaction with pharmacist consultations was assessed using the MISS-21J, following the scoring method described by Hanya et al. [19]. The MISS-21J includes four subscales: distress relief (six items), rapport (eight items), communication comfort (four items), and compliance intent (three items). Each item is rated on a 7-point scale, and subscale scores were calculated as the mean of item scores within each subscale. Therefore, each subscale score ranges from 1 to 7, with higher scores indicating greater satisfaction with the pharmacist consultation. In this study, the distress relief subscale was interpreted as a satisfaction domain reflecting patients’ perceived relief during the pharmacist consultation, rather than as a direct measure of clinical psychological distress. Because distress relief and rapport were considered particularly relevant to the early post-diagnosis consultation experience, these two subscales were prespecified as the primary outcomes. The remaining subscales were evaluated as secondary, exploratory outcomes.

## Sample size consideration

No formal a priori sample size calculation was performed specifically for the present analysis. The sample size was determined by the number of respondents who completed the nationwide web-based survey and met the eligibility criteria for this study. Therefore, the present analysis should be interpreted as an exploratory cross-sectional analysis based on the achieved analytic sample. As a post hoc reference, using a two-sample comparison as an approximation, the achieved group sizes of 175 and 232 provided approximately 80% power to detect a standardized between-group difference of approximately 0.42 under a two-sided significance threshold of 0.001. This sensitivity calculation was performed using G*Power version 3.1 and was used only to contextualize the achieved sample size, not to determine the sample size a priori.

### Statistical analysis

Participant characteristics were summarized according to pre-diagnosis pharmacist continuity (with vs. without). Inter-group comparisons were conducted using Fisher’s exact test for categorical variables and the Wilcoxon rank-sum test for continuous variables, as appropriate.

The primary analyses involved fitting multivariable linear regression models (ordinary least squares) with each MISS-21J subscale score treated as a continuous dependent variable and pre-diagnosis pharmacist continuity as the main independent variable. All models were adjusted for the following prespecified covariates: age at diagnosis (self-reported; continuous), sex, cancer type, residential area, parents available, partner status, and minor children. Adjusted means (least-squares means; LSMeans) and adjusted mean differences with 95% confidence intervals (CIs) were reported in Table 2.

For the two prespecified primary outcomes, distress relief and rapport, we used a conservative two-sided significance threshold of *p* < 0.001 as an interpretive criterion in this exploratory cross-sectional analysis. This threshold was chosen to provide a stringent criterion for the primary outcomes and was not intended to represent a formal Bonferroni or Holm adjustment. For the secondary outcomes, communication comfort and compliance intent, p-values were treated as nominal and interpreted as exploratory. All analyses were performed using JMP Pro 18 (SAS Institute, Cary, NC).

## Results

Participant characteristics are summarized in Table 1. The analysis included 407 AYA patients with an initial cancer diagnosis who reported having visited a community pharmacy and had contact with a pharmacist. Specifically, 175 (43.0%) patients were classified as having pre-diagnosis pharmacist continuity and 232 (57.0%) as not having such continuity. Among all 804 respondents with an initial cancer diagnosis, 175 (21.8%) met the definition of pre-diagnosis pharmacist continuity. This difference reflects the restriction of the primary analysis to respondents who used a community pharmacy after diagnosis. Regarding baseline characteristics, the two groups differed in terms of sex distribution and availability of parents, whereas age at diagnosis, residential area, cancer type, partner status, and presence of minor children were generally comparable (Table 1).

**Table 1:** Patient characteristics

Characteristic	Without pre-diagnosis pharmacist continuity (*n* = 232)	With pre-diagnosis pharmacist continuity (*n* = 175)	Total (*n* = 407)	*p* value
Sex, n (%)				0.002
Male	77 (33.2%)	86 (49.1%)	163 (40.0%)	
Female	155 (66.8%)	89 (50.9%)	244 (60.0%)	
Age				0.99
Mean ± SE	30.94 ± 0.45	30.93 ± 0.51		
Cancer type, n (%)				0.06
Gynecological cancer (Cervical/Endometrial/Ovarian)	67 (28.9%)	32 (18.3%)	99 (24.3%)	
Breast cancer	35 (15.1%)	27 (15.4%)	62 (15.2%)	
Stomach cancer	29 (12.5%)	26 (14.9%)	55 (13.5%)	
Colorectal cancer	16 (6.9%)	20 (11.4%)	36 (8.8%)	
Hematologic malignancies	18 (7.8%)	9 (5.1%)	27 (6.6%)	
Lung cancer	8 (3.4%)	15 (8.6%)	23 (5.7%)	
Thyroid cancer	13 (5.6%)	9 (5.1%)	22 (5.4%)	
Prostate cancer	8 (3.4%)	11 (6.3%)	19 (4.7%)	
Other	38 (16.4%)	26 (14.9%)	64 (15.7%)	
Area, n (%)				0.72
Hokkaido	7 (3.0%)	10 (5.7%)	17 (4.2%)	
Tohoku	21 (9.1%)	10 (5.7%)	31 (7.6%)	
Kanto	80 (34.5%)	58 (33.1%)	138 (33.9%)	
Chubu	44 (19.0%)	30 (17.1%)	74 (18.2%)	
Kinki	42 (18.1%)	35 (20.0%)	77 (18.9%)	
Chugoku	13 (5.6%)	10 (5.7%)	23 (5.7%)	
Shikoku	7 (3.0%)	4 (2.3%)	11 (2.7%)	
Kyushu	18 (7.8%)	18 (10.3%)	36 (8.8%)	
Parents available, n (%)				0.04
Yes	208 (89.7%)	144 (82.3%)	352 (86.5%)	
No	24 (10.3%)	31 (17.7%)	55 (13.5%)	
Partner available, n (%)				0.78
Yes	136 (58.6%)	105 (60.0%)	241 (59.2%)	
No	96 (41.4%)	70 (40.0%)	166 (40.8%)	
Minor children, n (%)				0.30
Yes	110 (47.4%)	92 (52.6%)	202 (49.6%)	
No	122 (52.6%)	83 (47.4%)	205 (50.4%)	

**Table 2:** Adjusted means and adjusted mean differences in MISS-21J subscale scores according to pre-diagnosis pharmacist continuity

Outcome (MISS-21J)	Adjusted mean (Without pre-diagnosis pharmacist continuity)	Adjusted mean (With pre-diagnosis pharmacist continuity)	Adjusted mean difference (With - Without)	95% CI	*p* value
Distress relief	4.72	5.33	0.61	0.42 to 0.81	< 0.001
Rapport	4.60	5.20	0.60	0.41 to 0.80	< 0.001
Communication comfort	3.73	3.27	-0.47	-0.74 to -0.18	0.002
Compliance intent	3.98	3.86	-0.12	-0.29 to 0.05	0.19

Adjusted for sex, age at diagnosis, cancer type, residential area, parents available, partner available, and minor children. Adjusted mean differences were calculated using the group without pre-diagnosis pharmacist continuity as the reference category

Table 2 presents the adjusted means (least-squares means; LSMeans) and adjusted mean differences estimated using multivariable linear regression models (ordinary least squares). Models were adjusted for age at diagnosis, sex, cancer type, residential area, parents available, partner status, and minor children. For the prespecified primary outcomes, patients with pre-diagnosis pharmacist continuity had higher scores in the MISS-21J domains of distress relief and rapport. The adjusted mean difference (with vs. without) was 0.61 for distress relief (95% CI, 0.42–0.81; *p* < 0.001) and 0.60 for rapport (95% CI, 0.41–0.80; *p* < 0.001) (Table 2).

Regarding the secondary outcomes, communication comfort was lower among patients with pre-diagnosis pharmacist continuity, with an adjusted mean difference of − 0.47 (95% CI, − 0.74 to − 0.18; *p* = 0.002) (Table 2). No clear inter-group difference was observed for compliance intent (adjusted mean difference, − 0.12; 95% CI, − 0.29 to 0.05; *p* = 0.19) (Table 2). The p-values for secondary outcomes were considered nominal (exploratory).

## Discussion

This study focused on the first community pharmacy visit shortly after AYAs received their initial cancer diagnosis. We analyzed the association between pre-diagnosis pharmacist continuity and patient-reported satisfaction with pharmacist consultations assessed using the MISS-21J. In covariate-adjusted analyses, patients with pre-diagnosis pharmacist continuity had higher scores in the MISS-21J domains of distress relief and rapport than those without such continuity.

AYAs typically face developmental stage–specific challenges related to education and employment, interpersonal relationships, fertility, and family formation [4–6]. Immediately after being diagnosed with cancer, they may experience a rapid increase in anxiety and uncertainty [22]. Considering this situation, it is not always easy to provide timely, patient-centered support. Having an established relationship with a pharmacist prior to diagnosis may lower the barrier to consultation and support the development of trust during pharmacy encounters [17]. Given this context, our findings suggest the potential value of continuity of pharmacist involvement in improving patients’ perceived consultation experience across the pre- and post-diagnosis period.

Communication comfort was lower among patients with pre-diagnosis pharmacist continuity. This unexpected finding should be interpreted cautiously because it emerged from an exploratory secondary analysis, and the present study was not designed to identify the underlying mechanisms; therefore, this finding should be regarded as hypothesis-generating. Although several explanations are possible, a closer pharmacist–patient relationship may facilitate more individualized conversations with increased opportunities to address personal background and sensitive topics [23]. From the perspective of autonomy and privacy, which are particularly important for AYAs, such interactions might have been perceived by some patients as less comfortable; however, this interpretation remains speculative [24, 25]. In addition, these patients may have higher expectations for pharmacists with whom they already have an established relationship, but these expectations may not always align with the reality of time-limited encounters at community pharmacies, potentially resulting in lower ratings of communication comfort [26]. In contrast, no clear inter-group difference was observed for compliance intent. Because the mechanisms underlying these secondary findings could not be examined in the present study, further research (e.g., AYA-sensitive communication design, longitudinal studies, and qualitative research) is needed to clarify how pharmacist involvement influences patients’ perceptions of communication and medication-taking behaviors.

One strength of this study is that it incorporated AYA-relevant social background factors (e.g., available parents, partner status, and the presence of minor children) as covariates. Although some baseline characteristics (e.g., sex and available parents) differed between groups, the association with the primary outcomes remained after covariate adjustment. Since family and social support resources may influence patient perceptions and experiences during cancer care in AYAs [27], adjusting for these factors supports the robustness of our findings.

This study also focused on the immediate post-diagnosis period at the first community pharmacy visit. However, the challenges and support needs of AYAs may change over the course of treatment [3, 9]. It would therefore be ideal to have a longer evaluation period that considers metastatic and recurrent phases. In adult cancer populations, pharmacist continuity has been associated with higher MISS-21J distress relief and rapport scores even during metastatic or recurrent disease [28]. Accordingly, it will be important to examine whether such continuity has similar or greater relevance among AYAs. Future studies should therefore clarify the optimal involvement of community pharmacists according to disease stage and treatment phase.

Several limitations should be noted. First, because this was a cross-sectional study, causal inference was not possible. Although the multivariable models adjusted for demographic, clinical, and social background variables, residual and unmeasured confounding may have remained. Factors such as healthcare-seeking behavior, prior patterns of community pharmacy use, treatment and prescription context, symptom burden, health literacy, and psychosocial background may have influenced both pre-diagnosis pharmacist continuity and patient-reported satisfaction with pharmacist consultations. Therefore, the observed findings should be interpreted as associations rather than causal effects of pharmacist continuity. Second, the possibility of misclassification could not be excluded because the data were self-reported. In addition, the exposure was based on self-reported continuity with the same pharmacist and did not verify formal registration under Japan’s family pharmacist system. Therefore, the findings should be interpreted primarily as evidence regarding continuity of pharmacist involvement rather than verified use of the formal system. Moreover, because the exposure was assessed using a single self-reported item, respondents classified as not having pre-diagnosis pharmacist continuity may have included individuals in different situations, such as those who did not have a family pharmacist before diagnosis and those who had one before diagnosis but were not supported by the same pharmacist at their first community pharmacy visit after diagnosis. Third, although the MISS-21J was originally validated in the context of over-the-counter medication counseling, the present study applied it to AYA patients with cancer in a prescription medication and cancer care context. Therefore, further validation of the MISS-21J in oncology and AYA-specific pharmacy consultation settings is warranted. Fourth, generalizability may be limited because the study used an online panel. Fifth, the analysis was restricted to patients who visited a community pharmacy after diagnosis. Accordingly, patients who did not use community pharmacies for various reasons, such as not receiving prescriptions soon after diagnosis or completing care through inpatient treatment or in-house dispensing, were not included. In addition, because the analysis was restricted to respondents who visited a community pharmacy and had pharmacist contact after diagnosis, the proportion classified as having pre-diagnosis pharmacist continuity should not be interpreted as the prevalence in all AYAs with cancer. Finally, the timing of the first post-diagnosis community pharmacy visit was based on respondents’ self-report and was not further operationally defined.

Despite these limitations, this study suggests that pre-diagnosis pharmacist continuity may be associated with more favorable patient-reported satisfaction with pharmacist consultations, particularly in the MISS-21J domains of distress relief and rapport, shortly after diagnosis among AYAs. Community pharmacists may therefore represent an accessible source of consultation for AYAs who engage with community pharmacy services. In this sense, the present findings may be interpreted less as evidence about a formal designation itself and more as evidence regarding the clinical value of continuity in pharmacist involvement experienced by patients in the current Japanese policy context.

## Conclusions

Among AYAs who were newly diagnosed with cancer, pre-diagnosis pharmacist continuity was associated with higher patient-reported satisfaction with pharmacist consultations, particularly in the MISS-21J domains of distress relief and rapport. A previously established and continuous relationship with a community pharmacist may be associated with a more favorable pharmaceutical care experience during the early post-diagnosis period.

## Supplementary Information

Below is the link to the electronic supplementary material.

Supplementary Material 1

## Data Availability

The datasets used and/or analyzed during the current study are available from the corresponding author on reasonable request.

## Abbreviations

AYA: Adolescents and young adults
MISS-21J: Japanese version of the Medical Interview Satisfaction Scale
CIs: Confidence intervals
