# Supplementary material for: Relationship of pre-diagnosis pharmacist continuity with patient-reported satisfaction at the first post-diagnosis community pharmacy visit among adolescents and young adults with cancer: a nationwide cross-sectional online survey
Source: J Pharm Health Care Sci. 2026 Jun 9;12:73. doi: 10.1186/s40780-026-00589-9 (PMC13479543; doi:10.1186/s40780-026-00589-9)
Supplement: Supplementary file 1 [file 40780_2026_589_MOESM1_ESM.docx]

**Supplementary Table 1 Survey item**

| Question content | | Item List |
| --- | --- | --- |
| Question A | Please rate your experience with the explanations and support you received from the pharmacist at the pharmacy on a scale of 1 to 7 when you first visited the pharmacy after your initial cancer diagnosis. | This item set was based on the MISS-21J study [19]. The MISS-21J consists of 21 items across four subscales: distress relief (6 items), communication comfort (4 items), rapport (8 items), and compliance intent (3 items). Each item was rated on a 7-point scale. Subscale scores were calculated as the mean of item scores within each subscale and ranged from 1 to 7, with higher scores indicating greater satisfaction with the pharmacist consultation. |
| Question B | Please indicate the primary cancer site at your initial cancer diagnosis. | Gynecological cancer (Cervical/Endometrial/Ovarian) |
|  |  | Breast cancer |
|  |  | Stomach cancer |
|  |  | Colorectal cancer |
|  |  | Thyroid cancer |
|  |  | Soft tissue tumor |
|  |  | Lung cancer |
|  |  | Prostate cancer |
|  |  | Malignant lymphoma |
|  |  | Leukemia |
|  |  | Esophageal cancer |
|  |  | Brain tumor |
|  |  | Liver cancer |
|  |  | Bone sarcoma |
|  |  | Urothelial carcinoma |
|  |  | Oral/Pharyngeal/Laryngeal cancer |
|  |  | Retinoblastoma |
|  |  | Hepatoblastoma |
|  |  | Germ cell tumor |
|  |  | Biliary tract cancer |
|  |  | Pancreatic cancer |
|  |  | Multiple myeloma |
|  |  | Other |
| Question C | Did you have a family pharmacist before your initial cancer diagnosis, and were you supported by the same family pharmacist at your first pharmacy visit shortly after the diagnosis? | Yes |
|  |  | No |
| Question D | What was your age (in years) at the time of your first cancer diagnosis? | 15–39 years  (1-year increments) |
| Question E | Do you have a parent who can provide support when needed? | Yes |
|  |  | No |
| Question F | Do you have a partner? | Yes |
|  |  | No |
| Question G | Do you have any minor children? | Yes |
|  |  | No |
